# Hypothetical Protein gene1038 Contributes to Colistin Resistance in Aeromonas hydrophila

**DOI:** 10.1128/AAC.01503-21

**Published:** 2021-11-17

**Authors:** Junqi Liu, Gang Xiao, Wangping Zhou, Jun Yang, Zhiliang Sun

**Affiliations:** a College of Veterinary Medicine, Hunan Agricultural University, Changsha, Hunan, China; b Veterinary Drug Laboratory, Hunan Institute of Animal and Veterinary Science, Changsha, Hunan, China; c Hunan Engineering Research Center of Veterinary Drugs, Changsha, Hunan, China

**Keywords:** *Aeromonas* spp., colistin resistance, gene1038

## LETTER

Inhibition of vital respiratory enzymes, such as NADH:ubiquinone oxidoreductase (complex I), type II NADH-quinone oxidoreductases (NDH-2), and malate:quinone oxidoreductase, in the inner membrane is a secondary antibacterial mechanism of colistin (1–3). However, colistin resistance mechanisms associated with this secondary mode of action of colistin have rarely been reported. Herein, we confirm that the hypothetical protein gene1038 was associated with colistin resistance in Aeromonas hydrophila by reducing antibiotic function in the inner membrane, providing novel knowledge on the generation of colistin resistance.

**FIG 1:**
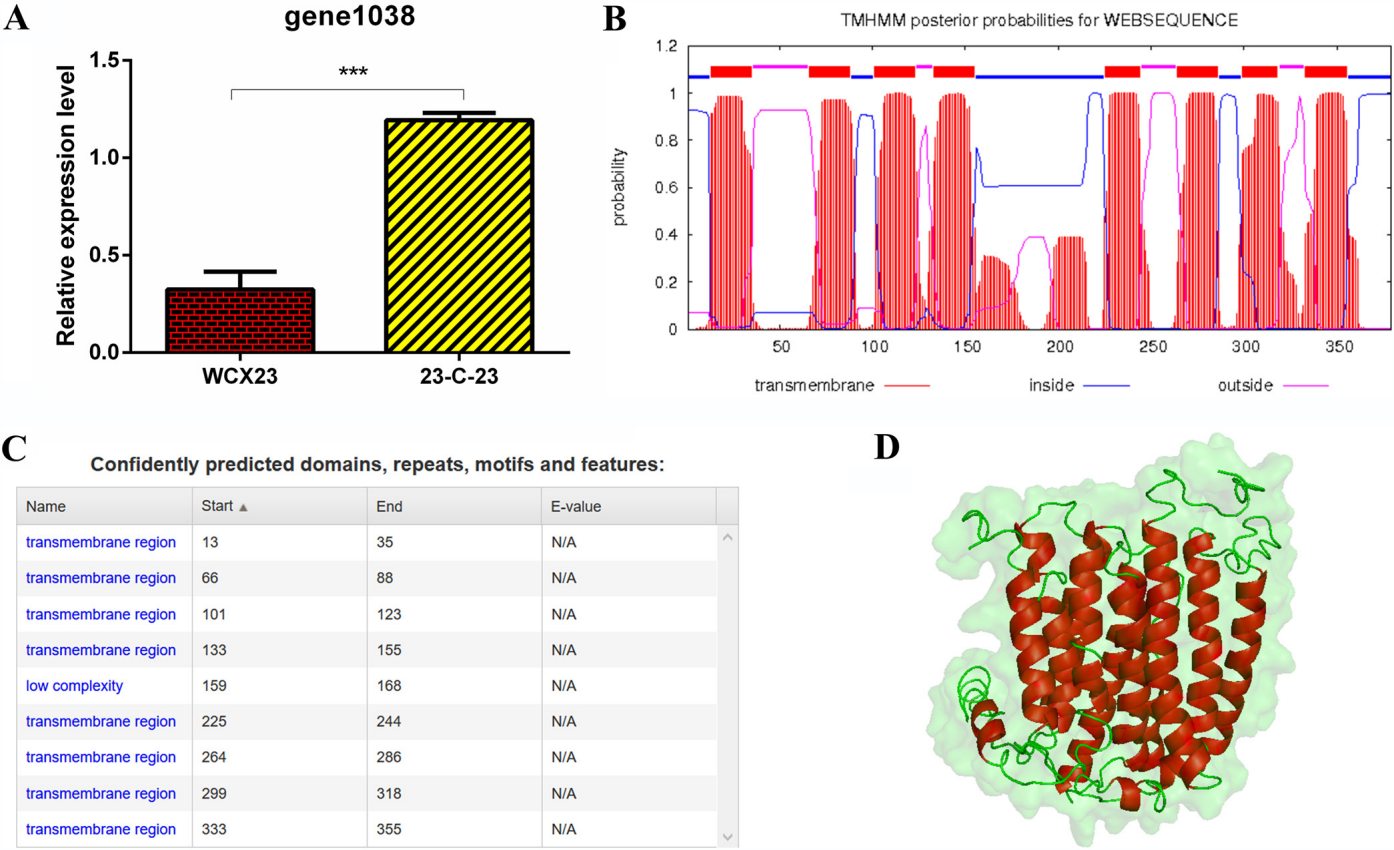
mRNA expression and protein modeling of gene1038. (A) Expression of gene*1038* in a colistin-susceptible strain (WCX23) and colistin-resistant strain (23-C-23). Relative expression levels of genes were determined using the 2^–ΔΔCT^ method (where CT is threshold cycle). Error bars represent the standard deviations from three biological replicates. Statistical analysis was performed using a Student two-tailed *t* test. ***, *P* value < 0.001. (B) TMHMM prediction results for gene*1038*. (C) SMART prediction results for gene*1038*. (D) I-Tasser homology modeling analysis of gene*1038*.

The expression of gene*1038* was significantly increased in the colistin-resistant strain 23-C-23 compared to that of the colistin-susceptible strain WCX23 (Fig. 1A) via quantitative reverse transcription-PCR (RT-PCR) using the primers gene*1038*-F (5′-GCTGCTTCGGCTTCCTCTAT-3′) and gene*1038*-R (5′-GGTCTCGCCGAACATGAGAT-3′) as previously described (4). This suggests that gene*1038* might be associated with colistin resistance in A. hydrophila. To test this hypothesis, gene*1038* was knocked out in a 23-C-23 background (23-C-23:Δgene*1038*) and subsequently restored in complemented strain 23-C-23:CΔgene*1038*, as previously described (4) (see Table S1 in the supplemental material). The results showed that the colistin MIC for 23-C-23:Δgene*1038* was 8-fold lower than for the parent strain and that the complementation of gene*1038* in 23-C-23:CΔgene*1038* restored resistance to colistin (Table 1). Furthermore, antimicrobial susceptibility testing indicated that 23-C-23, 23-C-23:Δgene*1038*, and 23-C-23:CΔgene*1038* showed similar resistance profiles toward other antimicrobial drugs (Table S2). These findings confirmed that gene*1038* is exclusively involved in colistin resistance.

**TABLE 1:** Colistin MICs of *A*. *hydrophila* strains

Strain	Description	Colistin MIC (mg/liter)
WCX23	*A. hydrophila* strain isolated from a snake with diarrhea (5)	1
23-C-23	WCX23 after 23 passages with colistin (4)	256
23-C-23:Δgene*1038*	23-C-23 with the gene*1038* knocked out	32
23-C-23:CΔgene*1038*	23-C-23:Δgene*1038* with gene*1038* complementation	256

gene*1038* had >97.36% similarity with hypothetical proteins in *A. hydrophila* and at least 86.81% similarity with other *Aeromonas* spp. via BLASTP analysis (https://blast.ncbi.nlm.nih.gov/Blast.cgi). In contrast, the maximum similarity with other species was only 66.23%. These results suggest that gene1038 is exclusive to *Aeromonas* spp. According to the predictions of the TMHMM server (http://www.cbs.dtu.dk/services/TMHMM/) and SMART (http://smart.embl.de/), the protein product of gene*1038*, as expected, forms eight transmembrane regions (Fig. 1B and C). Structural models of gene*1038* constructed using the i-TASSER server (https://zhanglab.ccmb.med.umich.edu/I-TASSER/) showed that the protein was structurally close to the membrane domain of respiratory complex I (Protein Data Bank accession no. 3RKO) (Fig. 1D), which was the target of colistin in the inner membrane (1). Accordingly, we speculated that upregulation of gene*1038* in *A. hydrophila* might offset the inhibition of complex I by colistin, leading to the impaired bactericidal action of colistin in the inner membrane.

**FIG 2:**
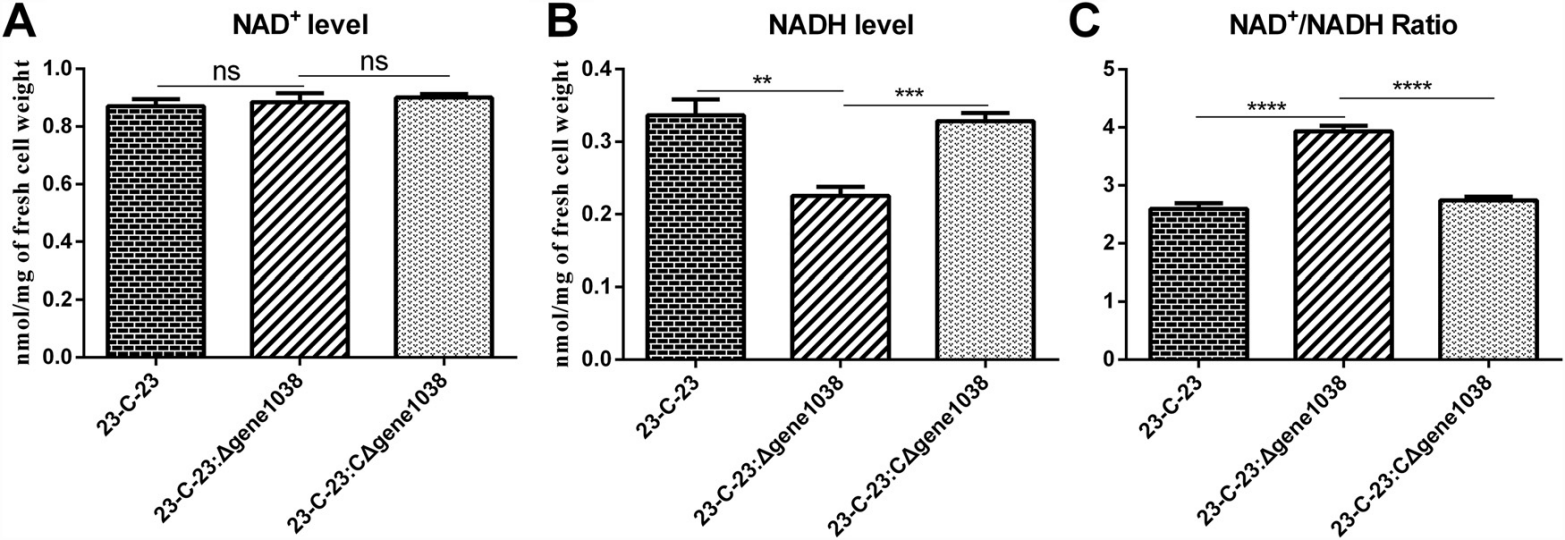
NAD^+^ and NADH levels and the NAD^+^/NADH ratio in bacterial strains. Changes in the NAD^+^ content (A), the NADH content (B), and the NAD^+^/NADH ratio (C) in 23-C-23, 23-C-23:Δgene*1038*, and 23-C-23:CΔgene*1038*. Error bars represent standard deviations from three biological replicates. Statistical analysis was performed using a Student two-tailed *t* test. **, *P* < 0.01; ***, *P* < 0.001; ****, *P* < 0.0001; ns, not significant.

We observed that gene*1038* was involved in NAD^+^/NADH ratio regulation using the NAD(H) level detection kit (Solarbio, Beijing, China) as reported previously (6). The deletion of gene*1038* was accompanied by a decrease in NADH concentration and an increase in the NAD^+^/NADH ratio (Fig. 2). The complementation of gene*1038* led to an increasing NADH concentration and a decreasing NAD^+^/NADH ratio (Fig. 2). These data indicated that gene*1038* participated in the upregulation of NADH, resulting in the downregulation of the NAD^+^/NADH ratio. We speculated that cells might utilize gene*1038* to regulate the NAD^+^/NADH ratio in response to the inhibition of complex I by colistin.

Conclusively, the hypothetical protein gene1038 is a protein exclusive to *Aeromonas* spp., it possesses 8 transmembrane regions, it is structurally close to the membrane domain of respiratory complex I, and it participates in the modulation of the NAD^+^/NADH ratio. We revealed a novel (to our knowledge) colistin resistance mechanism mediated by the upregulation of gene*1038* that might weaken colistin’s antibacterial effect through antagonizing the inhibition of respiratory complex I in the inner membrane.

Data were statistically analyzed using GraphPad Prism version 7.0 (GraphPad Software Inc., San Diego, CA, USA). The differences were analyzed using Student’s two-tailed unpaired *t* tests and are expressed as means ± standard deviations (SD), unless otherwise noted. Statistical significance was set at a *P *of <0.05.

### Data availability.

The nucleotide sequences of gene*1038* have been submitted to GenBank with the accession number MN862665.
