# Supplementary material for: Hypothetical Protein gene1038 Contributes to Colistin Resistance in Aeromonas hydrophila
Source: Antimicrob Agents Chemother. 2021 Nov 17;65(12):e01503-21. doi: 10.1128/AAC.01503-21 (PMC8597782; doi:10.1128/AAC.01503-21)
Supplement: Supplemental file 1 — Tables S1 and S2. Download aac.01503-21-s0001.pdf, PDF file, 0.08 MB [file aac.01503-21-s0001.pdf]

Supplementary data

Table S1 Primer sequences for gene knockout and complementation

	Primer sequences （5'→3'）	Description
**Gene deletion**		
gene*1038*-5F	CAGATATCTGCCGTAGTGCTCTTCTCG	For amplification of upstream homologous sequences of gene*1038*
gene*1038*-5R	GCTCCGGCATCATCTGCCAC	
gene*1038*-3F	GCTGATGCTCCTGCATTGGTTGG	For amplification of downstream homologous sequences of gene*1038*
gene*1038*-3R	CTGAGCCGACTCAATATCGATGTCAGC	
gene*103*8-CmF	GTGGCAGATGATGCCGGAGCGAGCTGCTTCGAA GTTCCTA	For amplification of the chloramphenicol resistance gene
gene*1038*-CmR	CCAACCAATGCAGGAGCATCAGCCATATGAATA TCCTCCTTAGTTCCTATTC	
**Gene complementation**		
gene*1038*-C-5F	CAGATATCTGCCGTAGTGCTCTTCTCG	For amplification of upstream homologous sequences containing gene1038
gene*1038*-C-5R	TAAACCGCCAAAACTATTACAAATCATGACAGC	
gene*1038*-C-3F	TCAGCCTTTCTGGCGGCC	For amplification of downstream homologous sequences
gene*1038*-C-3R	CAGCGACTTCCATGAGCTGTTCC	
gene*1038*-C-AprF	GCTGTCATGATTTGTAATAGTTTTGGCGGTTTAG GAATAGGAACTTATGAGCTCAGCCAA	For amplification of apramycin resistance gene
gene*1038*-C-AprR	GGCCGCCAGAAAGGCTGATAATGACCCCGAAGC AGGGTTATG	

Table S2 Antimicrobial susceptibility of strains

	23-C-23	23-C-23:Δgene*1038*	23-C-23:CΔgene*1038*
Amoxicillin *	4096; R	4096; R	2048; R
Lincomycin *	512; R	521; R	512; R
Streptomycin *	4096; R	>4096; R	4096; R
Spectinomycin *	4096; R	>4096; R	>4096; R
Kanamycin *	1024; R	512; R	1024; R
Gentamicin *	16; R	16; R	16; R
Azithromycin *	16; R	8; R	8; R
Cefradine *	512; R	512; R	256; R
Ofloxacin *	0.5; S	0.25; S	0.25; S
Ciprofloxacin *	0.5; S	0.5; S	0.5; S
Aztreonam*	1; S	1; S	1; S
Sulfamethoxazole*	256; S	256; S	256; S

S, susceptibility; I, intermediate; R, resistant.

* All units are in mg/L
